# Slow extension of the invading DNA strand in a D-loop formed by RecA-mediated homologous recombination may enhance recognition of DNA homology

**DOI:** 10.1074/jbc.RA119.007554

**Published:** 2019-04-11

**Authors:** Daniel Lu, Claudia Danilowicz, Tommy F. Tashjian, Chantal Prévost, Veronica G. Godoy, Mara Prentiss

**Affiliations:** From the ‡ Department of Physics, Harvard University, Cambridge, Massachusetts 02138,; § Department of Biology, Northeastern University, Boston, Massachusetts 02115, and; ¶ Laboratoire de Biochimie Théorique, CNRS UMR 9080, Institut de Biologie Physico-chimique (IBPC), Paris 75005, France

**Keywords:** DNA recombination, DNA polymerase, cooperativity, fluorescence resonance energy transfer (FRET), molecular dynamics, DNA damage, double-strand break (DSB), heteroduplex formation, RecA, strand displacement synthesis

## Abstract

DNA recombination resulting from RecA-mediated strand exchange aided by RecBCD proteins often enables accurate repair of DNA double-strand breaks. However, the process of recombinational repair between short DNA regions of accidental similarity can lead to fatal genomic rearrangements. Previous studies have probed how effectively RecA discriminates against interactions involving a short similar sequence that is embedded in otherwise dissimilar sequences but have not yielded fully conclusive results. Here, we present results of *in vitro* experiments with fluorescent probes strategically located on the interacting DNA fragments used for recombination. Our findings suggest that DNA synthesis increases the stability of the recombination products. Fluorescence measurements can also probe the homology dependence of the extension of invading DNA strands in D-loops formed by RecA-mediated strand exchange. We examined the slow extension of the invading strand in a D-loop by DNA polymerase (Pol) IV and the more rapid extension by DNA polymerase LF-*Bsu*. We found that when DNA Pol IV extends the invading strand in a D-loop formed by RecA-mediated strand exchange, the extension afforded by 82 bp of homology is significantly longer than the extension on 50 bp of homology. In contrast, the extension of the invading strand in D-loops by DNA LF-*Bsu* Pol is similar for intermediates with ≥50 bp of homology. These results suggest that fatal genomic rearrangements due to the recombination of small regions of accidental homology may be reduced if RecA-mediated strand exchange is immediately followed by DNA synthesis by a slow polymerase.

## Introduction

**Figure 1.**
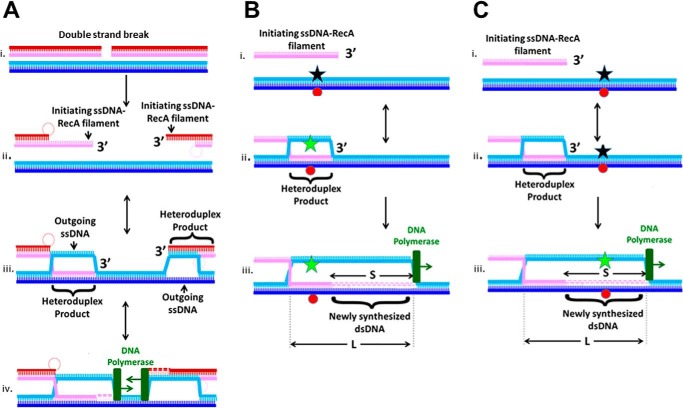
**Schematics of *in vivo* double-strand break repair and *in vitro* assays proving heteroduplex formation or extension of the invading strand in a D-loop.** *A*, schematic of *in vivo* repair. *Panel i*, initial double-strand break occurs in the DNA with the *red* and *pink* backbones. *Panel ii*, formation of ssDNA–RecA filaments on the ssDNA at the 3′ ends of the broken dsDNA. The illustration also shows degradation of the invading strands flanking the break, which occurs if the RecBCD pathway is followed. *Panel iii*, interactions between the ssDNA–RecA filaments and the unbroken dsDNA (*blue* strands) create heteroduplex dsDNA that pairs the invading and complementary strands, leaving the outgoing strand unpaired. *Panel iv*, after the heteroduplex reaches the 3′ end of the invading strand, the DNA polymerase can use the complementary strand as a template to extend the 3′ end of the invading strand. *B*, *panels i–iii*, *in vitro* monitoring of the heteroduplex product using FRET due to a fluorescein molecule (*star*) on the outgoing strand and a rhodamine molecule on the complementary strand (*red circle*). *Black stars* represent quenched fluorescein. *Green stars* indicate fluorescein molecules with no FRET. Formation of a heteroduplex product in the sequence region containing the fluorophores separates the outgoing and complementary strands, which increases fluorescein emission. The DNA polymerase is shown in *green*. It can perform strand displacement synthesis that extends the invading strand in the D-loop. *C*, *panels i–iii*, *in vitro* monitoring of the dsDNA structure beyond the 3′ end of the invading strand using FRET. Heteroduplex formation does not significantly increase emission, but strand displacement and DNA synthesis performed by the DNA polymerase can enhance emission.

During repair of breaks in double-stranded DNA (dsDNA), RecA-mediated recombination can create fatal rearrangements if the repair joins regions of accidental homology. In this work, we consider the possibility that the progression of DNA synthesis triggered by RecA-mediated recombination plays a role in rejecting such rearrangements. Fig. 1*A*, *panel i*, illustrates a double-strand break in the dsDNA with *red* backbones. On each side of the break, protein interactions form a single-stranded DNA (ssDNA)^2^ with a 3′ tail (see Fig. 1*A*, *panel ii*) (1). RecA subsequently binds to each ssDNA, forming two ssDNA–RecA filaments (1, 2). If the RecBCD pathway is followed, there is a gap between the sequences at the 3′ ends of the two filaments (3). To complete DNA repair, this gap must be filled by DNA synthesis (1).

The two ssDNA–RecA filaments catalyze alignment of the ssDNA with homologous sequences in dsDNA. If sufficient local homology is present, the base pairing of the complementary strand in the dsDNA is transferred from its original partner in the dsDNA to the invading ssDNA in the ssDNA–RecA filament. That transfer of base pairing is referred to as strand exchange. Strand exchange creates a heteroduplex dsDNA product in which the invading and complementary strands are base-paired (see Fig. 1*A*, *panel iii*) (1). After strand exchange, the complementary strand's original base pairing partner, the outgoing strand, remains unpaired, creating a D-loop (4). In the D-loop, the heteroduplex is surrounded by homoduplex dsDNA in which the outgoing and complementary strands remain base-paired. Once the heteroduplex product nears the 3′ end of the invading ssDNA, a DNA polymerase can extend the D-loop by adding bases to the 3′ end of the invading strand using the complementary strand as a template (Fig. 1*A*, *panel iv*) (1).

The extension of the invading strand in the D-loop depends on an interaction among a DNA polymerase, the 3′ end of the invading ssDNA, and the complementary strand in the dsDNA. The required geometry between the 3′ end of the invading ssDNA and the complementary strand can be established by heteroduplex strand exchange products near the 3′ end of the invading ssDNA (Fig. 1*A*, *panel iv*). Because heteroduplex formation depends on the homology between the invading and complementary strands (5–8), synthesis triggered by RecA-mediated homologous recombination (9) also depends on the homology between the invading and complementary strands. In the presence of synthesis, *L*, the total length of the dsDNA in which the invading and complementary strands are base-paired, is the sum of the length of the heteroduplex and the *S* bases in the synthesized dsDNA (see Fig. 1).

*In vivo* studies (10–12) have measured the dependence of the frequency of recombination on *N*, the number of contiguous homologous bp in a short region of homology. Those studies did not detect recombination when *N* was less than 20 bp (10–12). The measured frequency of recombination increased exponentially as *N* increased from 20 to 75 bp but only approximately linearly for *N* larger than ∼75 bp (11). Remarkably, the frequency of recombination for a sequence with *N* = 75 is ∼10 times larger than the frequency for a sequence with *N* = 50 (11).

*In vitro*, the stability of strand exchange products increases with *N* until approaching an asymptotic value at *N* = ∼20 (5, 7, 8, 13). In the absence of ATP hydrolysis, heteroduplex products with *N* ≥ 20 are very stable (13). This is not surprising because RecA is an ATPase (14), so hydrolysis occurs *in vivo* (15). With ATP hydrolysis, *in vitro* heteroduplex yield continues to increase slowly from *N* = 20 to *N* = 75, but even for *N* = 75 it does not represent complete product formation (13). Thus, the steep *N* dependence of *in vivo* incorporation (10–12) is not consistent with *in vitro* measurements of the *N* dependence of heteroduplex product lifetimes (8) or equilibrium product formation (13). Importantly, *in vitro* measurements of the influence of *N* on strand exchange products have not included DNA synthesis (5–8, 13, 16, 17).

If the RecBCD pathway is followed, strand exchange products are reversible until sufficient DNA synthesis has extended the invading strands in D-loops (13, 18, 19). Thus, the probability of incorporating an *N*-bp region of accidental homology into a genome may be influenced by the extension of the invading strand in the D-loop that is triggered by strand exchange.

Previous *in vitro* experiments have shown that *N* affects the triggering of DNA synthesis by RecA-mediated strand exchange (19). The previous studies indicated that heteroduplex products rarely trigger DNA synthesis unless the number of homologous bases is *N* ≥ 20 (19). However, that previous work did not consider how DNA synthesis that extends the invading strand in a D-loop affects the yield of heteroduplex products or how *N* influences the progress of invading strand extension after the extension is triggered by the formation of a heteroduplex product (19).

In this work, we extend that study to determine whether DNA synthesis that extends the invading strand in a D-loop affects the yield of heteroduplex products. We also probe how *N* influences the extension of the invading strand in the D-loop after DNA synthesis is initiated by RecA-mediated strand exchange. Many DNA polymerases can potentially perform DNA synthesis. In this work, we considered two different polymerases, *Bacillus subtilis* DNA polymerase, large fragment (DNA LF-*Bsu* Pol) and DNA polymerase (Pol) IV.

DNA Pol IV is up-regulated by the SOS response that follows formation of a double-strand break among other insults (20), and DNA Pol IV is known to extend invading strands in D-loops formed by RecA-mediated homologous recombination (9). Thus, we chose to consider how DNA Pol IV extends the invading strand in D-loops formed by RecA-mediated recombination *in vitro*.

When DNA synthesis does not require strand displacement, DNA Pol IV synthesis is inefficient (21), whereas synthesis by DNA LF-*Bsu* is rapid and processive (22). DNA LF-*Bsu* Pol is a well-characterized, commercially available protein that also provides rapid strand displacement DNA synthesis *in vitro*; therefore, we chose to compare invading strand extension performed by DNA Pol IV with the invading strand extension performed by DNA LF-*Bsu* Pol.

For either polymerase, we found that extension of the invading strand in a D-loop increases the yield of heteroduplex strand exchange products. Importantly, when the extension of the invading strand is limited to ≤15 nt, the fluorescence signals for both polymerases are similar. In contrast, when the invading strand can be extended by 50 or more nt, DNA LF-*Bsu* Pol extends the invading strand more than DNA Pol IV. We also found that the homology dependence of the extension of the invading strand in the D-loop by DNA Pol IV is quite different from the homology dependence of the extension by DNA LF-*Bsu* Pol. Finally, we present a model that could explain these differences in homology dependence and consider implications of the model for genomic rearrangements.

## Results

We used FRET to study both strand exchange and DNA synthesis that extends the invading strand in a D-loop formed by strand exchange. As in previous studies, a fluorescein label is placed on one of the dsDNA strands, and a rhodamine label is placed on the other strand (7, 13). Thus, initially rhodamine quenches the fluorescein label. Because the fluorophores are positioned on opposite strands in the dsDNA, the quenching becomes less efficient as the separation between the dsDNA strands increases.

Fig. 1*B* illustrates how heteroduplex formation can increase fluorescein emission if a FRET pair is placed within the region of the dsDNA that is homologous to the invading strand (6, 7). Thus, we monitored heteroduplex formation using FRET probes positioned within the region of the dsDNA that is homologous to the invading strand. Previous *in vitro* experiments studied the *N*-dependent stability of heteroduplex products in the absence of any DNA polymerase. If heteroduplex stability is influenced by the extension of the invading strand in a D-loop, then *in vitro* studies that do not include DNA polymerase may not capture the *N* probability of permanently incorporating regions of accidental homology into genomes. Thus, the influence of DNA polymerase could contribute to the large discrepancy between the *N* dependence of the *in vivo* (10–12) results and *N* dependence of *in vitro* (8, 13) results that did not include any DNA polymerase.

**Figure 2.**
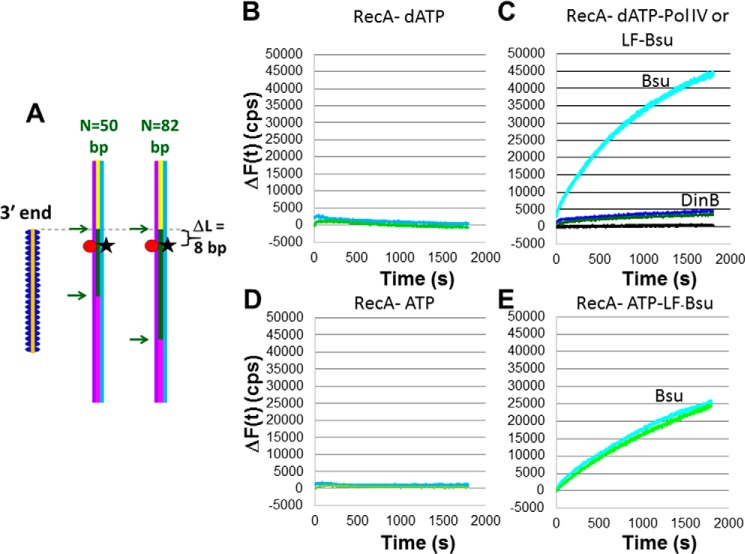
**Measurements of RecA-mediated heteroduplex product formation in the presence or absence of DNA synthesis.** *A*, schematic of the experimental setup with the initiating ssDNA shown in *orange*. The *purple* and *blue lines* indicate the complementary and outgoing strands in the dsDNA, respectively. The *green* regions in the dsDNA are sequence-matched to the corresponding regions in the 98-nt filament. The *yellow* regions in the 180-bp dsDNA extend beyond the 3′ end of the initiating strand, and the *magenta* regions are heterologous to the corresponding regions in the initiating strand. The *red circles* represent the rhodamine labels. The *black stars* represent fluorescein labels that are quenched when the complementary and outgoing strands are paired. The fluorophores are 58 (rhodamine) and 57 bp (fluorescein) from the end of the dsDNA. The 3′ end of the ssDNA–RecA filaments is 50 bp from the end of the dsDNA, yielding Δ*L* = 8. *B*, Δ*F*(*t*) *versus* time curves in the presence of RecA only and dATP for *N* = 82 (*light blue*) and *N* = 50 (*light green*). *C*, same as *B* but DNA Pol IV is also present for *N* = 82 (*dark blue*) and *N* = 50 (*dark green*), or LF-*Bsu* Pol is also present for *N* = 82 (cyan) and *N* = 0 (*black*). The estimated yield of ssDNA outgoing strand formation for LF-*Bsu* Pol and for DNA Pol IV is 80 and 6%, respectively. *D*, Δ*F*(*t*) *versus* time curves in the presence of RecA only and ATP for *N* = 82 (*light blue*) and *N* = 50 (*light green*). *E*, same as *D* but DNA LF-*Bsu* Pol is also present for *N* = 82 (*cyan*) and *N* = 50 (*bright green*). The estimated yield of ssDNA outgoing strand formation in the presence of LF-*Bsu* Pol is 45%. Results shown are for a single data set.

If extension of the invading strand in a D-loop enhances heteroduplex stability, then that extension should also enhance the yield of heteroduplex strand exchange products. Fig. 2*A* shows a schematic of experiments designed to test whether the extension of the invading strand in a D-loop increases the yield of heteroduplex products. It is a particular example of the general scheme illustrated in Fig. 1*B*.

All of the experimental results were obtained using the same 180-bp dsDNA. The dsDNA interacted with 98-nt ssDNA–RecA filaments that were long enough (98 nt) to allow for complete filament formation. Because the dsDNA was always the same, we controlled *N*, the number of contiguous bp at the 3′ end of the invading strand, by changing the sequences of the 98-nt invading strands. The remaining bases in the invading strand were not sequence-matched to the corresponding bases in the complementary strand. In Fig. 2*A*, the matching sequences are indicated by the *green* regions in the dsDNA. The ends of the homologous regions are indicated by the *green arrows*.

The fluorophores are 58 (rhodamine) and 57 bp (fluorescein) from the end of the dsDNA, so *D*_fluor_ ∼ 58 bp is the separation between the fluorescent labels and the end of the dsDNA. The 3′ end of the ssDNA–RecA filaments is 50 bp from the end of the dsDNA. We will refer to that separation as *D*_init3′_, and we define Δ*L* = *D*_fluor_ − *D*_init3′_. Fig. 2*A* shows that Δ*L* = 8 bp, so when Δ*L* > 0, the labels are positioned within the region of the dsDNA containing the *N* homologous bases (the homologous region).

In these experiments, we measure the number of counts per second recorded by a fluorometer. We then calculate Δ*F*(*t*), the difference between the number of counts obtained at time *t* = 0 when the invading ssDNA is heterologous to the dsDNA (*N* = 0) and the number of counts measured at a time *t*.

Fig. 2, *B* and *D*, show that in the absence of a DNA polymerase, the values of the Δ*F*(*t*) curves are quite small. The presence of either polymerase significantly increases the Δ*F*(*t*) signals if all dNTPs are present. Experiments that use 90-bp dsDNA also show that either polymerase increases the Δ*F*(*t*) signals if all dNTPs are present (Fig. S1). For both polymerases, when filaments are prepared in dATP, the fluorescence signal is larger than the fluorescence signal obtained when the filaments are prepared with ATP (Fig. 2, *C* and *E*). Importantly, experiments using 180-bp dsDNA indicate that in either buffer the increase in fluorescence for DNA LF-*Bsu* Pol is much larger than the increase in fluorescence for DNA Pol IV.

The differences between the results for the two polymerases might be a consequence of differences in the initiation of the extension of the invading strand in the D-loop. To test whether the two polymerases initiate invading strand extension at different rates, we performed experiments in which the homoduplex tail is preserved for both polymerases because the polymerases only extend the invading strand by 1 base. Fig. S2 shows results of experiments with fluorophores that are 11 (rhodamine) and 9 bp (fluorescein) from the end of the dsDNA, and the 3′ end of the initiating strand is 11 bp from the end of the dsDNA. Rhodamine modifies a “T” base, and for this 90-bp dsDNA in the presence of dATP only 1 nucleotide can be incorporated (23). Fig. S2 shows that either polymerase can extend the invading strand by binding dATP to the rhodamine-labeled nucleotide. Furthermore, the fluorescence signals are similar for both polymerases.

To test whether the extensions for the two polymerases are different when the invading strand can be extended by more than 15 nt, we conducted additional experiments. Fig. 1*C* illustrates how the extension of the invading strand in a D-loop can increase fluorescein emission if a FRET pair is placed beyond the 3′ end of the invading strand. In the absence of synthesis, the invading strand does not include any bases in this region; therefore, in the absence of synthesis, strand exchange cannot form a heteroduplex product in this region.

**Figure 3.**
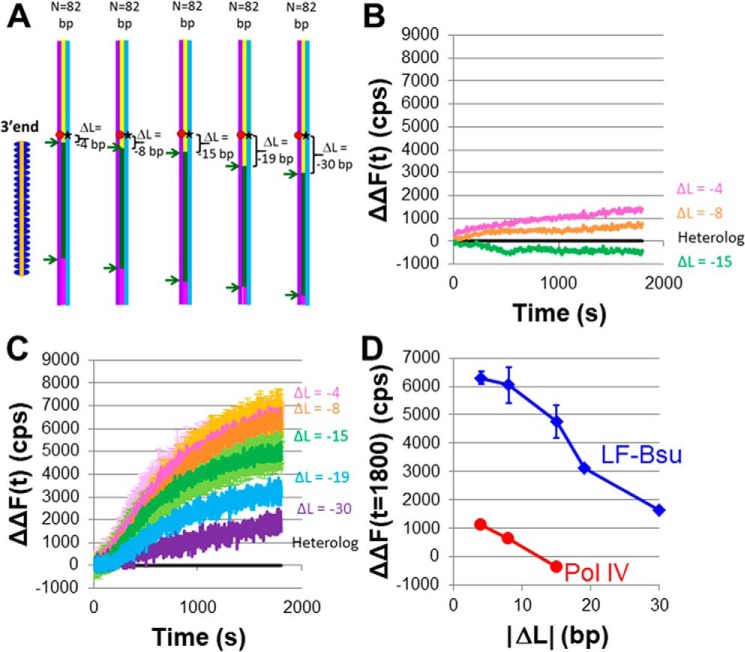
**Measurements of invading strand extension in a D-loop formed by RecA-mediated heteroduplex product formation when *N* = 82.** *A*, schematic of the experimental design using the same symbols as in Fig. 2*A*. The dsDNA is 180 bp long, and the fluorophores are 58 (rhodamine) and 57 bp (fluorescein) from the end of the dsDNA; the 3′ end of the ssDNA–RecA filaments is 62, 66, 73, 77, or 88 bp from the end of the dsDNA, yielding Δ*L* values of −4, −8, −15, −19, and −30, respectively. *B*, ΔΔ*F*(*t*) *versus* time curves averaged over 20 s for strand exchange in the presence of DNA Pol IV for Δ*L* = −4 (*magenta*), Δ*L* = −8 (*orange*), and Δ*L* = −15 (*green*). The second Δ indicates that the Δ*F*(*t*) curves for the heterologous ssDNA–RecA filament (*i.e. N* = 0) were subtracted from the Δ*F*(*t*) curves for *N* > 0. The yield of outgoing strand formation is 2.5 and 1.5% for Δ*L* = −4 and Δ*L* = −8, respectively. *C*, analogous results in the presence of DNA LF-*Bsu* Pol for Δ*L* = −4 (*magenta*), Δ*L* = −8 (*orange*), Δ*L* = −15 (*green*), Δ*L* = −19 (*blue*), and Δ*L* = −30 (*purple*). The *dark* lines correspond to averages over several data sets, and the corresponding *error bars* are shown in *lighter colors*. The yield of outgoing strand formation is between 12.3 (Δ*L* = −4) and 3.3% (Δ*L* = −30). *D*, ΔΔ*F*(*t* = 1800) *versus* Δ*L* curves corresponding to *B* (*red*) and Fig. 3*C* (*blue*). *Error bars* represent S.D. for multiple independent data sets.

Fig. 3*A* is a particular example of the general scheme shown in Fig. 1*C*. All of the experiments shown in Fig. 3 used the same 180-bp dsDNA that was used in Fig. 2, but *N* = 82 for all of the data shown in Fig. 3. We varied the position of the 3′ end of the ssDNA–RecA filaments so that *D*_init3′_ = 62, 66, 73, 77, or 88 bp from the end of the dsDNA (Fig. 3). Each *D*_init3′_ corresponds to a different Δ*L*, where Δ*L* is the separation between the fluorophores and the 3′ end of the ssDNA. By comparing results for different Δ*L* values, we can probe the progress of the extension of the invading strand in the D-loop created by the heteroduplex product. In Fig. 3*A*, Δ*L* = *D*_fluor_ − *D*_init3′_ = −4, −8, −15, −19, or −30 bp.

**Figure 4.**
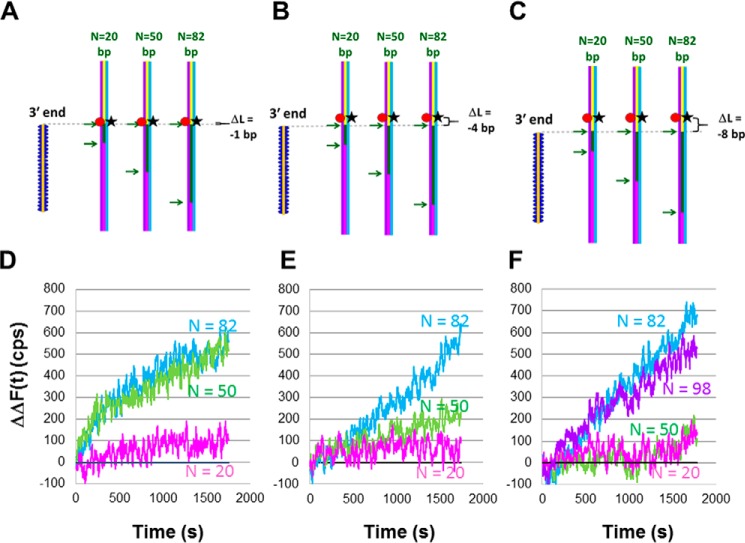
**Measurements of the *N* dependence of invading strand extension in a D-loop resulting from RecA-mediated heteroduplex product formation when DNA synthesis is performed by DNA Pol IV.** *A*, schematic of the experimental design using the same symbols as in Fig. 2*A*. The dsDNA is 180 bp long, and the fluorophores are 58 (rhodamine) and 57 bp (fluorescein) from the end of the dsDNA. The 3′ end of the ssDNA–RecA filaments is 59 bp from the end of the dsDNA, yielding Δ*L* = −1. *B*, same as *A* but the 3′ end of the invading strands is 62 bp from the end of the dsDNA, yielding Δ*L* = −4. *C*, same as *A* but the 3′ end of the invading strands is 66 bp from the end of the dsDNA, yielding Δ*L* = −8. *D*, ΔΔ*F*(*t*) *versus* time curves averaged over 20 s for *N* = 82 (*blue*), *N* = 50 (*green*), *N* = 20 (*magenta*), and *N* = 0 (*black*) for the experimental design shown in *A*. The second Δ indicates that the fluorescence signal of the heterologous ssDNA–RecA filament (*N* = 0) has been subtracted from each curve. The results are for a single data set. *E*, ΔΔ*F*(*t*) *versus* time curves for *N* = 82 (*blue*), *N* = 50 (*green*), *N* = 20 (*magenta*), and *N* = 0 (*black*) for the experimental design shown in *B. F*, ΔΔ*F*(*t*) *versus* time curves for *N* = 98 (purple), *N* = 82 (*blue*), *N* = 50 (*green*), *N* = 20 (*magenta*), and heterologous *N* = 0 (*black*) are shown for the experimental design shown in *C*. Analogous curves without the subtraction of the heterologous filament are shown in Fig. S6. Results are for a single data set. The yield of outgoing strand formation is about 1% for *N* = 82 in all three cases.

**Figure 5.**
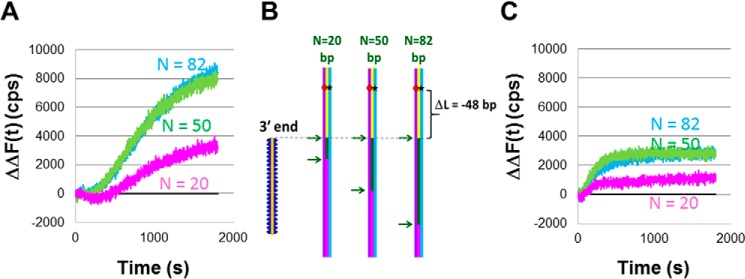
**Measurements of the *N* dependence of invading strand extension in a D-loop resulting from RecA-mediated heteroduplex product formation when DNA synthesis is performed by DNA LF-*Bsu* Pol.** *A*, ΔΔ*F*(*t*) *versus* time curves for Δ*L* = −1 and for *N* = 82 (*blue*), *N* = 50 (*green*), *N* = 20 (*magenta*), and *N* = 0 (*black*). The experimental schematic is shown in Fig. 4*A*. The yield of outgoing strand formation is 14% for *N* = 82 and 50 and 6% for *N* = 20. *B*, schematic of the experimental design using the same symbols as in previous figures. The dsDNA is 180 bp long, and fluorophores are positioned 11 (rhodamine) and 9 bp (fluorescein) from the end of the dsDNA. The 3′ end of the invading strands is 59 bp from the end of the dsDNA, yielding Δ*L* = −48. *C*, ΔΔ*F*(*t*) *versus* time curves for Δ*L* = −48 and *N* = 82 (*blue*), *N* = 50 (*green*), *N* = 20 (*magenta*), and *N* = 0 (*black*). The yield of outgoing strand formation is ∼5% for *N* = 82 and 50 and ∼2% for *N* = 20.

Fig. S3 shows Δ*F*(*t*) curves for Δ*L* = −1 obtained for strand exchange in the absence of DNA Pol IV. Those very small signals might represent transient DNA melting at the 3′ end of the invading strand, consistent with the dsDNA structure in the molecular models shown in Fig. S4. Comparison of the results in the presence and absence of the DNA polymerases suggests that the fluorescence increases shown in Figs. 3–5 are not dominated by effects resulting solely from heteroduplex formation but instead dominantly report on the action of the polymerases.

In the presence of either polymerase, the Δ*F*(*t*) fluorescence signals for the heterologous ssDNA–RecA filaments are not negligible. The Δ*F*(*t*) curves for DNA Pol IV and DNA LF-*Bsu* Pol are shown in Fig. S5, *A* and *B*, respectively. For each DNA polymerase, the same Δ*F*(*t*) curve for heterologous ssDNA applies to all of the Δ*L* values; therefore, consistent with previous work (19), in Fig. 3 for each Δ*L* value we show a graph of ΔΔ*F*(*t*), the difference between the Δ*F*(*t*) curve for *N* = 82, and the Δ*F*(*t*) curve for a heterologous filament.

Fig. 3*B* shows the ΔΔ*F*(*t*) results for DNA Pol IV. In the figure, the *magenta*, *orange*, and *green* curves show results for Δ*L* = −4, −8, and −15, respectively. Fig. 3*C* shows the ΔΔ*F*(*t*) *versus* time results for DNA LF-*Bsu* Pol in which the *magenta*, *orange*, *green*, *blue*, and *purple* curves show results for Δ*L* = −4, −8, −15, −19, and −30 bp, respectively. Fig. 3*C* indicates that DNA synthesis by DNA LF-*Bsu* Pol can extend at least 30 bp. Fig. 3*D* shows the ΔΔ*F*(1800) values as a function of Δ*L* for DNA Pol IV (*red circles*) and DNA LF-*Bsu* Pol (*blue diamonds*).

Because Fig. 3 shows that during 1800 s the extension of the invading strand by DNA LF-*Bsu* Pol can progress to at least 30 bp, we pursued additional experiments to probe how much further the extension of the invading strand might progress. Fig. S6A shows a schematic of an experimental design in which the 3′ ends of the invading strands are 62 and 77 bp from the end of the dsDNA with *D*_fluor_ = 11, yielding Δ*L* = −51 and Δ*L* = −66, respectively. Fig. S6, *B* and *C*, show Δ*F*(*t*) and ΔΔ*F*(*t*) curves, respectively, for the system shown schematically in Fig. S6A. The data in Fig. S6 indicate that synthesis by DNA LF-*Bsu* Pol can sometimes extend beyond 51 bp, although the Δ*F*(*t*) curve for Δ*L* = −66 just barely exceeds the results for the heterologous filament. These results are consistent with previous measurements, suggesting that the processivity of DNA LF-*Bsu* Pol is 1–55 nt (22). In sum, the results in Figs. 2 and 3 suggest that DNA LF-*Bsu* Pol extends the invading strand in a D-loop more than DNA Pol IV and that heteroduplex product formation increases as the extension of the invading strand increases.

*In vivo*, dsDNA synthesis will almost never reach the end of a dsDNA, so in what follows we will highlight results with *D*_init3′_ > ∼55 bp. We chose *D*_init3′_ ≥ 55 because Fig. 3 and Fig. S6 indicate that *D*_init3′_ ∼ 55 is sufficiently large that during our observation time RecA-triggered synthesis by DNA Pol IV will never reach the end of the dsDNA and synthesis by DNA LF-*Bsu* Pol will almost never reach the end of the dsDNA.

Having shown that heteroduplex product formation and the extension of the invading strand in a D-loop differ for LF-*Bsu* Pol and DNA Pol IV, we now consider whether the homology dependence of the extension of the invading strand in a D-loop is different for the two polymerases. Fig. 4, *A–C*, show the schematic representations for a system with *D*_fluor_ = 58 bp and *D*_init3′_ = 59, 62, and 66 bp, respectively. Those *D*_init3′_ values correspond to Δ*L* = −1, −4, and −8 bp, respectively.

The Δ*F*(*t*) curves obtained with DNA Pol IV are shown in Figs. S7, *A–C*, for Δ*L* = −1, Δ*L* = −4, and Δ*L* = −8, respectively. In each figure, the *magenta*, *green*, and *blue* curves show the results for *N* = 20, 50, and 82, respectively. Fig. 4, *D–F*, show the ΔΔ*F*(*t*) curves obtained with DNA Pol IV and all dNTPs for Δ*L* = −1, Δ*L* = −4, and Δ*L* = −8, respectively. The data shown have been averaged over 20 s. Data for Δ*L* = −2 is shown in Fig. S8. For the data with Δ*L* = −1 and Δ*L* = −2, the results for *N* = 82 and *N* = 50 are very similar, but for Δ*L* = −4 and Δ*L* = −8, the results for *N* = 82 and *N* = 50 differ. Importantly, for Δ*L* = −8, the *N* = 50 signal is indistinguishable from the signal for *N* = 20 or the signal for the heterologous filament. Thus, the *N* dependence of synthesis increases strongly as we monitor fluorescence at positions more distant from the 3′ end of the filament.

Fig. 4 shows that the extension of the invading strand in a D-loop by DNA Pol IV depends strongly on *N*. To determine whether this strong *N* dependence applies to all polymerases, we repeated the experiments using DNA LF-*Bsu* Pol. Fig. 5*A* shows ΔΔ*F*(*t*) obtained with DNA LF-*Bsu* Pol for Δ*L* = −1. These data are analogous to the results for DNA Pol IV that are shown in Fig. 4*D*, so the schematic shown in Fig. 4*A* also applies to Fig. 5*A*. For both polymerases, the signals for *N* = 50 and *N* = 82 are similar when Δ*L* = −1; therefore, when Δ*L* = −1, neither polymerase distinguishes between the two *N* values.

Fig. 5*B* shows the schematic for Δ*L* = −48 bp, and Fig. 5*C* shows the resulting ΔΔ*F*(*t*) curves. Fig. S9 shows the Δ*F*(*t*) results corresponding to ΔΔ*F*(*t*) curves shown in Fig. 5. For DNA LF-*Bsu* Pol, the ΔΔ*F*(*t*) curves for Δ*L* = −1 and Δ*L* = −48 (Fig. 5, *A* and *C*) are quite different, suggesting that for DNA LF-*Bsu* Pol not all of the strand displacement synthesis that reaches Δ*L* = −1 also reaches Δ*L* = −48; however, in both cases, the result for *N* = 82 is the same as the result for *N* = 50. Thus, the results shown in Fig. 5 indicate that even when the extension of the invading strand in a D-loop is as large as 50 bp, the probability that DNA LF-*Bsu* Pol will extend the invading strand is insensitive to *N* for *N* ≥ 50. In contrast, Fig. 4 shows that for DNA extension as small as 4 bp, the probability that DNA Pol IV will extend the DNA is sensitive to *N* even when *N* ≥ 50.

To provide graphical representations of the differences between the *N* dependences of the synthesis performed by the two polymerases and to offer some comparison with *in vivo* results, we quantified the *N* dependence of the DNA synthesis based on the fluorescence signals that we observed after 1800 s. In particular, we considered interactions with Δ*L* < 0, and we defined the ΔΔ*F*(1800) ratio [*N*] as the ratio of the ΔΔ*F*(1800) value for a given *N* to the ΔΔ*F*(1800) value for *N* = 82. Thus, by definition, the ΔΔ*F*(1800) ratio [82] = 1.

**Figure 6.**
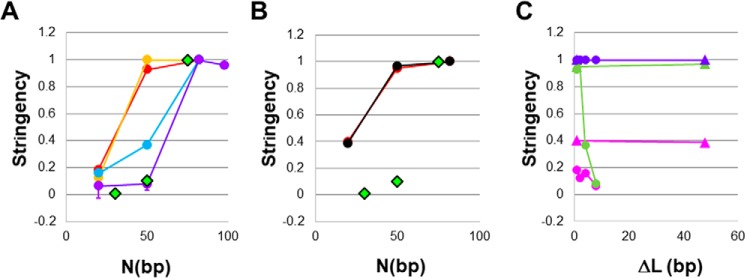
***N* or |Δ*L*| dependence of the ΔΔ*F*(1800) values shown in Figs. 4, S7, and 5, normalized to the result for *N* = 82 (ΔΔ*F*(1800) ratio [*N*]).** *A*, for DNA Pol IV, stringency = ΔΔ*F*(1800) ratio [N] as a function of *N* for fluorescent labels with different separations from the 3′ end of the original initiation ssDNA. The *red*, *orange*, *blue*, and *purple* curves show results for labels at Δ*L* = −1, −2, −4, and −8 bp, respectively. The *green diamonds* show the stringency for *in vivo* studies (11). *B*, same as *A* but for DNA LF-*Bsu* Pol, showing results for Δ*L* = −1 (*red*) and Δ*L* = −48 (*black*). *C*, the data are the same as the data shown in *A* and *B*, but the ΔΔ*F*(1800) ratio [*N*] values for particular *N* are plotted as a function of |Δ*L*|. The *circles* and *triangles* represent the results for DNA Pol IV and DNA LF-*Bsu* Pol, respectively. The colors of the curves correspond to the *N* values. The *magenta*, *green*, and *blue* lines represent *N* = 20, 50, and 82, respectively. For DNA LF-*Bsu* Pol the ΔΔ*F*(1800) ratio [*N*] values are insensitive to |Δ*L*|, but for DNA Pol IV the results depend strongly on |Δ*L*|. *Error bars* represent S.D. values.

Fig. 6*A* shows ΔΔ*F*(1800) ratio [*N*] *versus N* for the experimental results using DNA Pol IV that are shown in Fig. 4 and Fig. S8. The *red*, *orange*, *blue*, and *purple* curves correspond to Δ*L* = −1, −2, −4, and −8 bp, respectively. Fig. 6*A* shows that ΔΔ*F*(1800) ratio [50] ∼ 1 when Δ*L* = −1 or −2, but ΔΔ*F*(1800)ratio [50] decreases as Δ*L* becomes more negative. To compare the *in vitro* results with *in vivo* results, we have presented the *in vivo* results in the same graph.

*In vivo* experiments measured the probability that a region of accidental homology containing *N* homologous bases will be incorporated in a genome. To compare our *in vitro* results with results of those *in vivo* studies (11), we determined the probability that a sequence including *N* = 82 bp would be incorporated into a genome. We then normalized the *in vivo* results for all other *N* values by dividing the measured probability for a particular *N* value to the probability for *N* = 82 (*green diamonds*) (11). Although the *red* and *orange* lines depart strongly from the *green diamonds*, the *purple* curve is similar to the *green diamond* result, indicating that for Δ*L* = −8, the ΔΔ*F*(1800) ratio [82] *versus N* is consistent with the *N* dependence of the *in vivo* ratios. Thus, for DNA Pol IV, the *N* dependence of DNA extension by a few bp is not consistent with *in vivo* results, but the *N* dependence of extending DNA by ∼8 bases is.

In contrast to the results for DNA Pol IV shown in Fig. 6*A*, Fig. 6*B* indicates that for DNA LF-*Bsu* Pol, the *N* dependence of the extension of the invading strand in a D-loop is not consistent with *in vivo* results even when the synthesis extends over ∼50 bp. Fig. 6*C* combines the results shown in Fig. 6, *A* and *B*, but each curve corresponds to a given *N* value, and the *x axis* represents the |Δ*L*| value. Thus, by following a single curve, one can visualize how the *N*-dependent discrimination changes as the separation between the fluorescent labels and the 3′ end of the invading strand increases. The results for DNA LF-*Bsu* Pol are all straight lines, indicating that the *N* dependence is insensitive to |Δ*L*| for Δ*L* < 0. By definition, for *N* = 82, the curve for DNA Pol IV is also a straight line; however, for lower *N* values, the curves for DNA Pol IV decrease rapidly as the separation between the fluorescent labels and the 3′ end of the invading strand increases.

## Discussion

**Figure 7.**
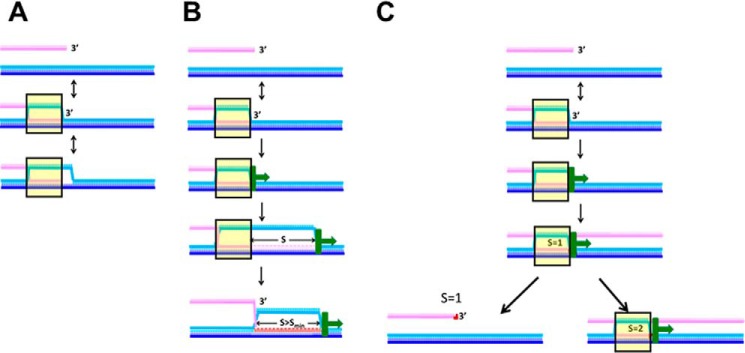
**Illustrations showing how the speed of D-loop extension could influence the *N* dependence of the extension of the invading strand in a D-loop created by RecA-mediated formation of a heteroduplex product.** The invading, complementary, and outgoing strands are shown in *pink*, *dark blue*, and *light blue*, respectively. Regions in which all three DNA strands are bound to a RecA nucleoprotein filament are indicated by the *yellow rectangles* with *black outlines*. The DNA polymerase synthesis is represented in *green*. The direction of polymerization is indicated by the *green arrow*, and the newly synthesized extension of the invading strand is shown in *red. A*, case in which no polymerase is present, so extension of the invading strand in a D-loop due to strand displacement DNA synthesis is not possible. Without synthesis, all products remain reversible. Some transient melting of the dsDNA may occur near the 3′ end of the invading strand. *B*, case in which the extension of the invading strand in a D-loop is so rapid that every heteroduplex that includes *N* > *N*_min_ and reaches the 3′ end of the invading strand creates an irreversible product by triggering DNA synthesis that progresses forever even if the heteroduplex partially reverses on the 5′ side of the invading strand. Thus, once the D-loop forms, it never completely collapses. This system cannot distinguish between *N* values ≥*N*_min_. If *N* < *N*_min_, then *S* remains less than *S*_min_, so the D-loop can completely collapse. Thus, the system can distinguish between *N* values if *N* < *N*_min_. *C*, case in which *S* is always less than *S*_min_. Thus, the D-loop can always completely collapse, and extension is always influenced by the behavior of the heteroduplex because synthesis proceeds slowly enough that the heteroduplex product reverses before the polymerase synthesizes *S*_min_ bases. The *black arrow* pointing to the *lower left* indicates a case in which only 1 base is synthesized before the D-loop collapses. The *arrow* pointing to the *lower right* shows a case in which a second base can be synthesized before the D-loop collapses. The time required for the heteroduplex to collapse depends on *N*, so *S* increases with *N*. After the D-loop collapses, restarting synthesis may require formation of another heteroduplex.

In this work, we studied the *in vitro* extension of invading strands in D-loops formed by RecA-mediated recombination and compared results for DNA LF-*Bsu* Pol with results for DNA Pol IV. Our observations suggest that DNA synthesis by either polymerase stabilizes heteroduplex products (Figs. 2 and S1) and that both polymerases initiate invading strand synthesis at a similar rate (Figs. S1 and S2); however, during our 1800-s observation time, DNA LF-*Bsu* Pol extends the invading strand more than DNA Pol IV if the invading strand can be extended by more than 15 nt (Fig. 3). Furthermore, for DNA LF-*Bsu* Pol, the rapid extension of the invading strand is similar for regions of accidental homology that include over >50 bp (Figs. 5 and 6). In contrast, for DNA Pol IV, regions of accidental homology that include 82 bp trigger more extensive invading strand extension than regions of accidental homology that include only 50 bp (Figs. 4 and 6). These differences in the homology dependence of invading strand extension are consistent with a model suggesting that slow addition of successive bases to the invading strand provides additional kinetic proofreading steps that help discriminate against regions of accidental homology extending over >50 bp, whereas rapid extension does not offer the same discrimination (Fig. 7). Thus, slow initial extension of the invading strand in D-loops formed by RecA-mediated recombination may play an important role in suppressing genomic rearrangement associated with heteroduplex products formed by regions of accidental homology that include more than 50 bp.

Figs. 2 and S1 suggest that the extension of the invading strand in D-loops triggered by RecA-mediated recombination stabilizes heteroduplex products. Thus, *in vitro* measurements of the homology dependence of heteroduplex stability performed in the absence of DNA synthesis may not capture the homology dependence governing incorporation of a region of accidental homology into a genome.

Fig. 1 shows that if the heteroduplex exists, then extending the invading strand increases *L*, the total length of the dsDNA in which the complementary and invading strands are paired. This increase in *L* could underlie the synthesis-dependent increase in heteroduplex yield that is shown in Figs. 2 and S1 as we discuss in the following. Figs. S1 and S2 show that for the 90-bp dsDNA the heteroduplex yields for both polymerases are almost identical. We propose the results are very similar because both polymerases initiate synthesis at a similar rate, and synthesis by either polymerase can remove the ∼15-bp homoduplex tail at the end of the 90-bp dsDNA. As a result, the *L* values for the two polymerases are the same for the conditions in Figs. S1 and S2 that limit invading strand extension, *S*, to <15 bp. In contrast, Fig. 3 shows that for the 180-bp dsDNA, construct *S* frequently exceeds 15 bp when the invading strand is extended by DNA LF-*Bsu* Pol; therefore, *L* values for DNA LF-*Bsu* Pol are much larger than for DNA Pol IV. Fig. 2 shows that for the 180-bp dsDNA, the heteroduplex yields for DNA LF-*Bsu* Pol are much greater than for DNA Pol IV. Thus, we propose that Fig. 2 shows different results for the two polymerases because heteroduplex stability increases with *L*. Furthermore, we speculate that this general result can be extended to other polymerases or to more complex systems.

Double-strand break repairs that erroneously incorporate regions of accidental homology into genomes pose a serious threat to bacteria because bacterial genomes contain many long repeated sequences (19) that can produce genomic rearrangement if regions of accidental homology create strand exchange products (24). Previous work has suggested that RecA-mediated homologous recombination uses multistep kinetic proofreading to discriminate against strand exchange products involving accidental homology (25–28). The stringency offered by a kinetic proofreading system can be enhanced by adding subsequent kinetic proofreading steps (29). In what follows, we will discuss the differences between the results for the two polymerases and consider implications of those differences for *in vivo* incorporation of sequences with *N* contiguous homologous bp.

Fig. 6 suggests for DNA LF-*Bsu* Pol that the *S* values for *N* = 50 and *N* = 82 are the same, but for DNA Pol IV, the *S* value for *N* = 82 is larger than the *S* value for *N* = 50. Interestingly, Fig. 6 indicates that the homology dependence that governs 8 bp of synthesis by DNA Pol IV *in vitro* is similar to the homology dependence that governs incorporation of sequences into genomes.

Fig. 7 illustrates a very simple model that could explain this result. The model assumes that the stability of heteroduplex products depends on *N*. Consistent with previous experimental results (19), we assume that extension of the invading strand in a D-loop can only be triggered if the heteroduplex extends very close to the 3′ end of the invading strand. The model also assumes that until *S* exceeds *S*_min_ the extension of the invading strand in the D-loop is promoted by the continued presence of a heteroduplex product; however, after *S* exceeds *S*_min_, the extension of the invading strand in the D-loop no longer depends on the presence of a heteroduplex product.

Fig. 7*A* represents the case in which there is no polymerization at all. This was the situation in previous *in vitro* studies of the *N*-dependent stability of heteroduplex products (5–8). This case is not relevant *in vivo*.

Fig. 7*B* illustrates a case in which extension of the invading strand in the D-loop is so rapid that *S* can exceed *S*_min_ when heteroduplex products include *N* > *N*_min_. Those *N*_min_ bases are required for the heteroduplex to live long enough for D-loop extension to exceed *S*_min_. For smaller *N* values, the heteroduplex lifetime is so short that *S* does not exceed *S*_min_ even though the extension of the invading strand is very rapid. Thus, extension of the invading strand in the D-loop remains sensitive to *N* when *N* < *N*_min_, but is insensitive to *N* for *N* > *N*_min_. Furthermore, for *N* > *N*_min_, DNA synthesis would continue forever, so for *N* > *N*_min_ the fluorescence would be independent of Δ*L*.

If these conditions applied to our experiments, then for all *N* > *N*_min_ the fluorescence curves shown in Figs. 4 and 5 should be independent of Δ*L* and approach the asymptotic value corresponding to complete product formation. This is not true for either polymerase; however, for DNA LF-*Bsu* Pol, the stringency of the strand exchange is independent of Δ*L* for *N* ≥ 50. This suggests that although synthesis by DNA LF-*Bsu* Pol does not continue forever, the extension of the invading strand in the D-loop is insensitive to *N* for *N* ≥ 50, consistent with the model's predictions for rapid extension of the invading strand in a D-loop if *N*_min_ < 50.

Fig. 7*C* represents an intermediate case between Fig. 7, *A* and *B*. In this case, during the observation time the increase in *S* is so slow that *S* always remains less than *S*_min_. As a result, synthesis cannot progress unless a heteroduplex product is present. In addition, if each initiation of synthesis is considered a step in kinetic proofreading, then a distributive polymerase provides additional kinetic proofreading steps. If this were true, the number of kinetic proofreading steps required to reach the fluorophores would increase as Δ*L* becomes more negative. Thus, as Δ*L* becomes more negative, the Δ*F*(*t*) signals for lower *N* values should decrease more rapidly than the Δ*F*(*t*) signals for higher *N* values. This proposal is consistent with the results for DNA Pol IV that are shown in Fig. 4.

Thus, the experiments suggest that it is advantageous for RecA-mediated homologous recombination to be immediately followed by strand displacement DNA synthesis by an especially inefficient polymerase rather than synthesis by a polymerase that can efficiently extend the invading strand in the D-loop. This could explain the previously puzzling *in vivo* results hinting that DNA Pol IV usually performs the synthesis triggered by RecA-mediated homologous recombination (30) even though DNA Pol IV synthesis is inefficient and error-prone (21). This suggestion is also consistent with *in vitro* studies showing that DNA Pol IV can extend D-loops formed by RecA, but DNA Pol V cannot (9). Importantly, the error rate for DNA Pol IV is <10^−2^–10^−3^ (21). Thus, that error rate may not be very deleterious if only ∼8 bp must be synthesized before DNA Pol IV is replaced by a DNA polymerase capable of rapidly performing strand displacement synthesis of more than 1 kb of DNA that is typically removed when the RecBCD pathway is followed (3).

In this work, we have only considered *in vitro* interactions that include ssDNA–RecA filaments, dsDNA, and either DNA LF-*Bsu* Pol or DNA Pol IV. We do not argue that the *in vivo* interaction necessarily involves DNA Pol IV, and we do not discount the possibility that other proteins play important roles in double-strand break repairs that follow the RecBCD pathway; however, slow extension of the invading strand in D-loops may provide a previously unsuspected stringency-enhancing intermediate step between RecA-mediated homologous recombination and the rapid and accurate synthesis required to complete the invading strand bases removed by RecBCD.

## Experimental procedures

### Strand exchange and DNA synthesis assay

To recreate DNA recombination and synthesis reactions *in vitro*, we mixed 0.06 μm 98-nt ssDNA–RecA filament with 0.06 μm labeled dsDNA and finally added DNA polymerase. We used 90- or 180-bp dsDNAs and ssDNAs of varying lengths of homology (from the heterologous *N* = 0 up to *N* = 98) and a total length of 98 nt. For polymerase, we used either 1 μm *Escherichia coli* DNA Pol IV (obtained using Pol IV overproducer plasmids (31, 32)) or 5 units of DNA LF-*Bsu* Pol (New England Biolabs; 5000 units/ml). DNA Pol IV reactions were done in RecA buffer containing 0.1 mg/ml BSA, 2 mm dATP, and 0.4 mm dNTPs, whereas DNA LF-*Bsu* Pol experiments were performed in RecA buffer containing 1 mm ATP and 30 mm NaCl.

The ssDNA–RecA filaments were prepared by mixing the 0.06 μm ssDNA with 2 μm RecA (New England Biolabs), 1 mm ATP or dATP, 10 units/ml pyruvate kinase, and 3 mm phosphoenolpyruvate (ATP regeneration system) and with 0.2 μm single-stranded binding protein, all in RecA buffer (70 mm Tris-HCl, 10 mm MgCl_2_, and 5 mm DTT, pH 7.6). This mixture was incubated at 37 °C for 10 min.

### Preparation of dsDNA samples

The short 90-bp labeled dsDNA was obtained by heating a mixture of the corresponding oligonucleotides to 90 °C and then cooled back to 40 °C at each 1 °C per step and equilibrated for 1 min at each step. The fluorescein emission was followed at 518 nm at each temperature step using excitation at 493 nm.

To prepare the long construct, 180-bp labeled dsDNA, a 90-nt ssDNA containing an internal rhodamine label on base 58 (or base 11) from the 5′ end and a 5′ end–phosphorylated oligonucleotide (82 bases) containing an internal fluorescein label (position 57 or 9 from the 3′ end) were first annealed together. Then, a 90-base 5′ end–phosphorylated oligonucleotide was annealed with a 98-base oligonucleotide to produce another dsDNA but without labels. The two dsDNAs (labeled and unlabeled) were annealed and ligated overnight at 16 °C with T4 DNA ligase in ligase reaction buffer (50 mm Tris, 10 mm MgCl_2_, 1 mm ATP, and 10 mm DTT, pH 7.5; New England Biolabs). The 180-bp construct was purified by running a 3% agarose gel in Tris borate/EDTA buffer for 2 h (6 V/cm). The 180-bp band was visualized with a midrange UV transilluminator and cut. Finally, the dsDNA was extracted from the agarose using a Nucleospin kit (Machery and Nagel, Bethlehem, PA). The oligonucleotide sequences used to prepare the 90- and 180-bp dsDNAs as well as the 98-nt ssDNA for the ssDNA–RecA filaments are all listed in Tables S1–S3.

### FRET measurements

To measure the fluorescence signal of these strand exchange reactions, the ssDNA–RecA filament, dsDNA, dNTPs, and polymerase mixture was placed in a quartz cuvette. Using FluorEssence spectroscopy software and the automated FluoroMax spectrofluorometer (Horiba, Edison, NJ), the emission of the fluorescein label was read by using a 2-nm slit and 493-nm excitation wavelength. The fluorescence emission in counts per second (cps) was detected at 518 nm with a 2-nm slit. The reaction was run for 30 min with emission measurements every 1 s and an integration time of 0.5 s. Temperature was kept constant at 37 °C.

To calibrate for the formation of free ssDNA outgoing strand, measurements of dilutions of the corresponding ssDNA containing fluorescein were performed. The fluorescence signal in cps was considered equivalent to 100% yield of free outgoing ssDNA formation. The signals at 1800 s in all of the experiments were compared with the signal for 100% yield to calculate the corresponding yields.

## Author contributions

D. L., C. D., T. F. T., C. P., and M. P. data curation; D. L., C. D., C. P., V. G. G., and M. P. writing-original draft; C. D. and M. P. conceptualization; C. D. and M. P. supervision; C. D., T. F. T., and M. P. methodology; C. P., V. G. G., and M. P. formal analysis.

## Supplementary Material

Supporting Information

## Footnotes

2 The abbreviations used are:

ssDNA: single-stranded DNA
Pol: polymerase
nt: nucleotide(s)
LF: large fragment.
