# Supplementary material for: Slow extension of the invading DNA strand in a D-loop formed by RecA-mediated homologous recombination may enhance recognition of DNA homology
Source: J Biol Chem. 2019 Apr 11;294(21):8606–16. doi: 10.1074/jbc.RA119.007554 (PMC6544866; doi:10.1074/jbc.RA119.007554)
Supplement: Supporting Information [file supp_294_21_8606__index.html]

Slow extension of the invading DNA strand in a D-loop formed by RecA-mediated homologous recombination may enhance recognition of DNA homology — D-loop extension rates influence homology recognition — Slow extension of the invading DNA strand in a D-loop formed by RecA-mediated homologous recombination may enhance recognition of DNA homology — D-loop extension rates influence homology recognition — Supporting Information 

# Slow extension of the invading DNA strand in a D-loop formed by RecA-mediated homologous recombination may enhance recognition of DNA homology

## Supporting Information

- Supporting Information (to be published online) - Supporting information
