# Supplementary material for: Slow extension of the invading DNA strand in a D-loop formed by RecA-mediated homologous recombination may enhance recognition of DNA homology
Source: J Biol Chem. 2019 Apr 11;294(21):8606–16. doi: 10.1074/jbc.RA119.007554 (PMC6544866; doi:10.1074/jbc.RA119.007554)
Supplement: Supporting Information [file supp_RA119.007554_143131_2_supp_314017_ppt7rc.docx]

**Supplementary Information**

**Slow extension of the invading DNA strand in a D-loop formed by RecA-mediated homologous recombination may enhance recognition of DNA homology**

Daniel Lu, Claudia Danilowicz, Tommy F. Tashjian, Chantal Prévost, Veronica G. Godoy, and Mara Prentiss

**This file includes 3 Supplementary Tables and 9 Supplementary Figures.**


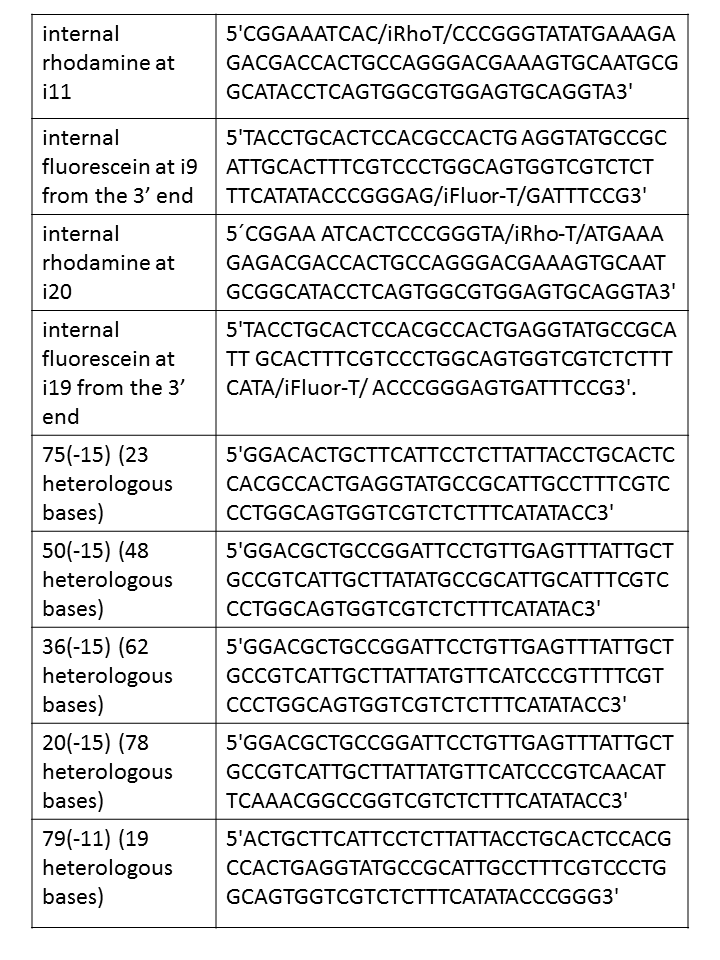


**Table S1. Oligonucleotides used for 90 bp dsDNA preparation and filaments.** Oligonucleotides for filaments that interact with the dsDNA are named by their homology length N and ΔL = D_fluor_ - D_init3′_. Thus for example 75(5) indicates the oligonucleotide for a filament with N = 75 and ΔL = 5 (D_init3′_ = 15). The lowercase “i” is used to indicate the position of a label from the end of the dsDNA that extends beyond the 3′ end of the initiating strand when the sequence matched regions of the initiating strand and the dsDNA are aligned, so e.g. i19 means 19 bases from the end of the dsDNA.

**
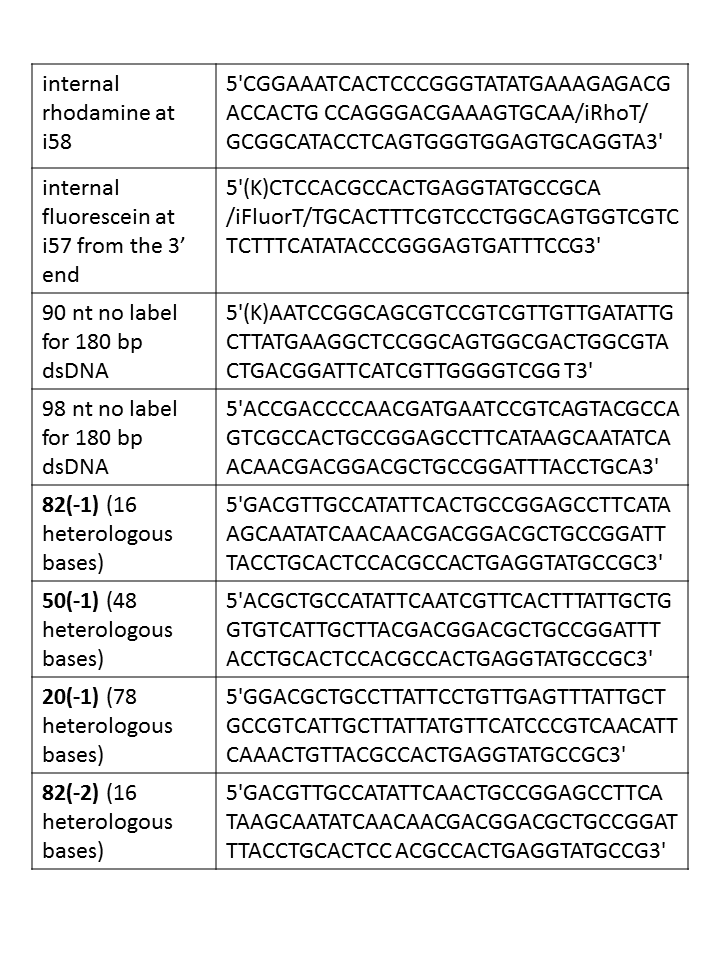
**

**Table S2. Oligonucleotides for 180 bp dsDNA preparation with internal rhodamine 58 bases from the 5′ end and internal fluorescein 57 bases from the 3′ end and oligonucleotides for experiments.**

**
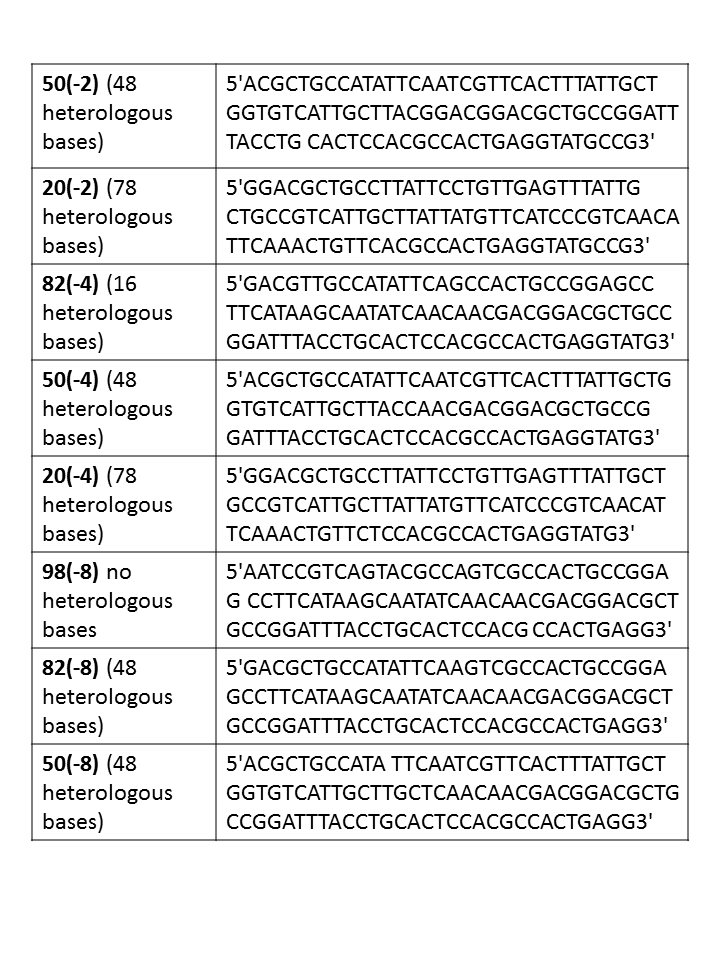
**

**Table S2 continued**

**
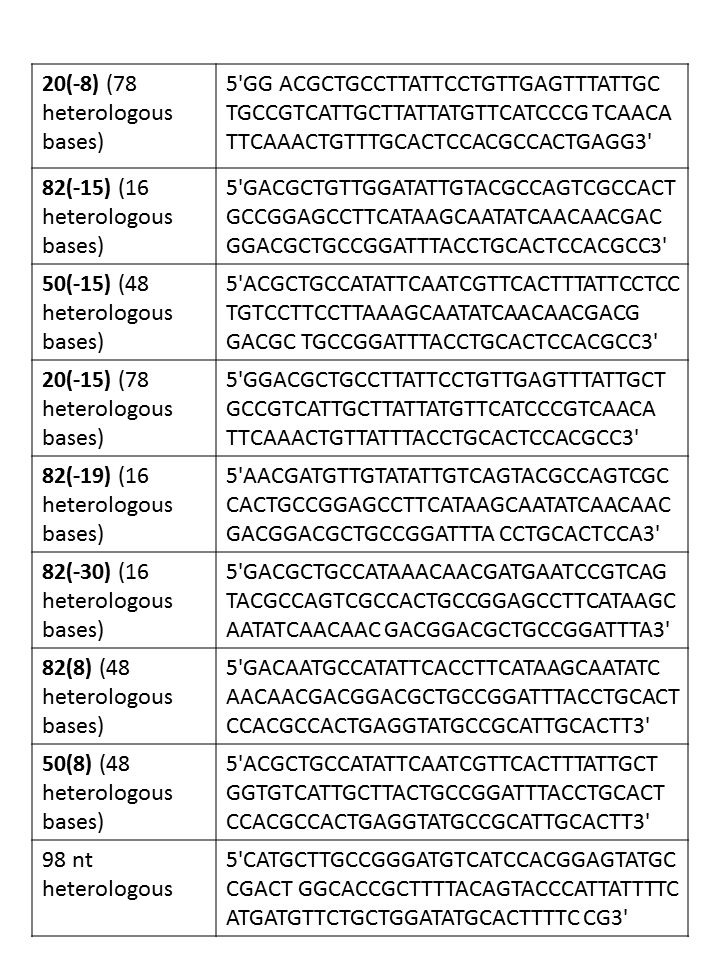
**

**Table S2 continued**

**
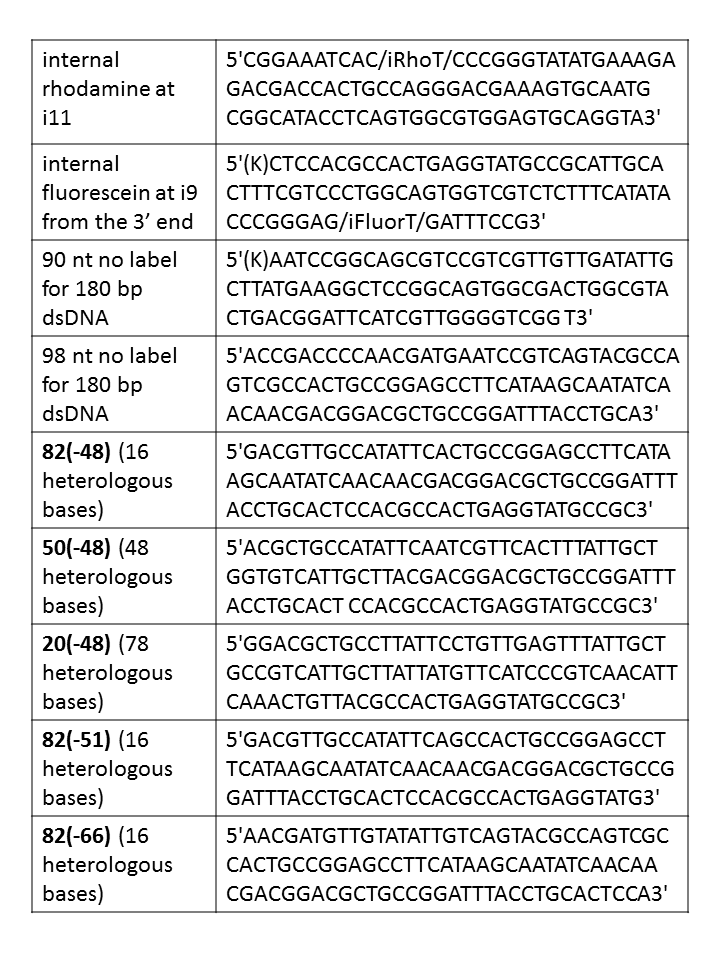
**

**Table S3. Oligonucleotides for 180 bp dsDNA preparation with internal rhodamine 11 bases from the 5′ end and internal fluorescein 9 bases from the 3′ end and oligonucleotides for experiments.**


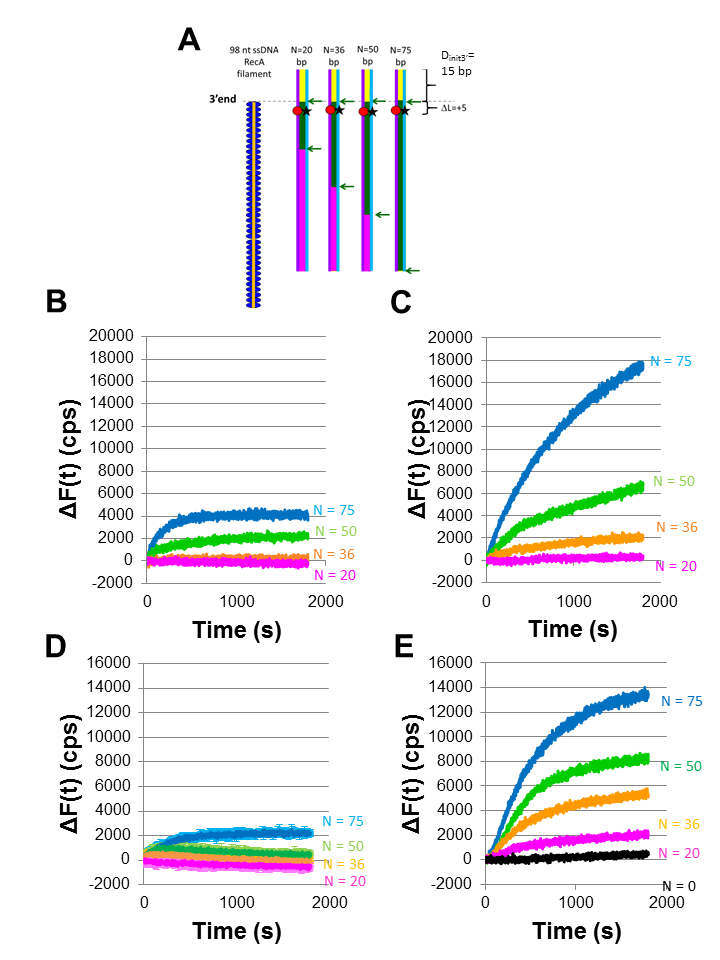


**Figure S1. FRET based measurements of heteroduplex product formation with and without DNA Pol IV polymerase or DNA LF-Bsu Pol.** A, schematic of the experimental design showing a 98 nt ssDNA-RecA filament, with the initiating ssDNA shown in orange. The color scheme is the same as in Fig. 2A. The fluorophores are 20 (rhodamine) and 19 bp (fluorescein) from the end of the dsDNA, and the 3′ end of the initiating strands is 15 bp from the end of the dsDNA, yielding ΔL = 5. Thus, the fluorophores are positioned within the dsDNA region that is homologous to the initiating strands. B, ΔF(t) vs. time curves in the presence of RecA only for N = 75 (blue), N = 50 (green), N = 36 (orange), and N = 20 (magenta). The results shown are for one data set. C, analogous curves in the presence of DNA Pol IV and all dNTPs. D, RecA only results for the buffer used with DNA LF-Bsu Pol. E, same as C, but in the presence of DNA LF-Bsu Pol. Result for the heterologous filament is shown in black. For this short (90 bp) dsDNA the ΔF(t) signals associated with the two polymerases may be similar because either polymerase can extend the invading strand to the end of the dsDNA.


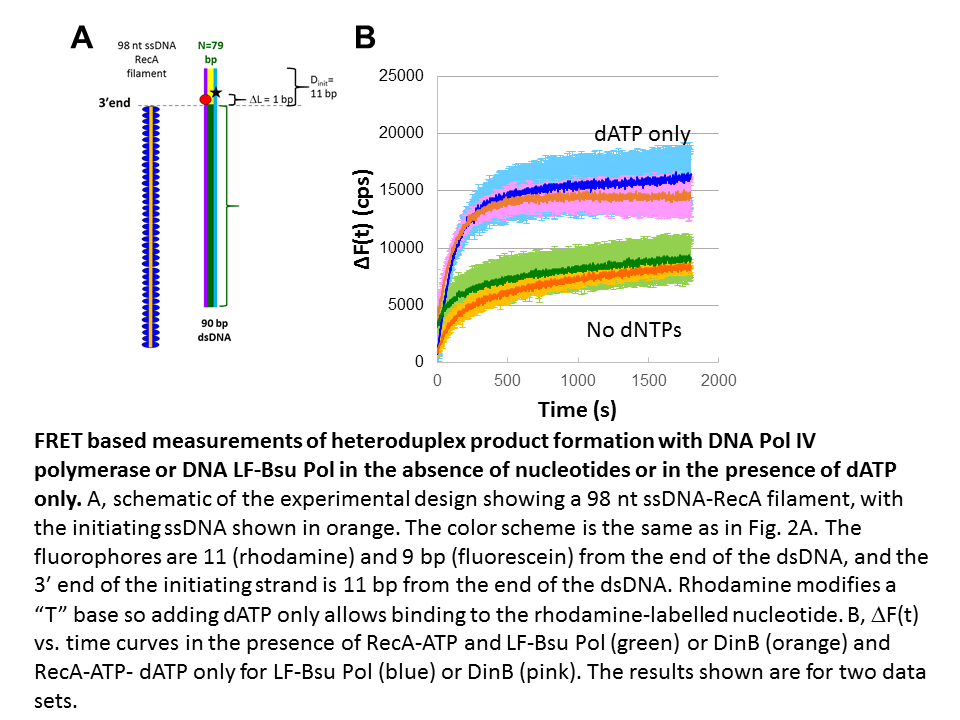


**Figure S2. FRET based measurements of heteroduplex product formation with DNA Pol IV polymerase or DNA LF-Bsu Pol in the absence of nucleotides or in the presence of dATP only.** A, schematic of the experimental design showing a 98 nt ssDNA-RecA filament, with the initiating ssDNA shown in orange. The color scheme is the same as in Fig. 2A. The fluorophores are 11 (rhodamine) and 9 bp (fluorescein) from the end of the dsDNA, and the 3′ end of the initiating strand is 11 bp from the end of the dsDNA. Rhodamine modifies a “T” base so adding dATP only allows binding to the rhodamine-labelled nucleotide. B, ΔF(t) vs. time curves in the presence of RecA-ATP and LF-Bsu Pol (green) or DinB (orange) and RecA-ATP- dATP only for LF-Bsu Pol (blue) or DinB (pink). The results shown are for two data sets.

**Figure S3. FRET based measurements of conformation disruptions beyond the 3′ end of the ssDNA-RecA filaments.** ΔF(t) vs. time curves for N = 82 (blue), N = 50 (green) and N = 20 (magenta). Schematic of the experimental design is the same as the schematic shown in Fig. 4A, so these results are analogous to those in Fig. 4D but without the presence of DNA Pol IV. The label is 1 bp beyond the 3′ end of the initiating strand.


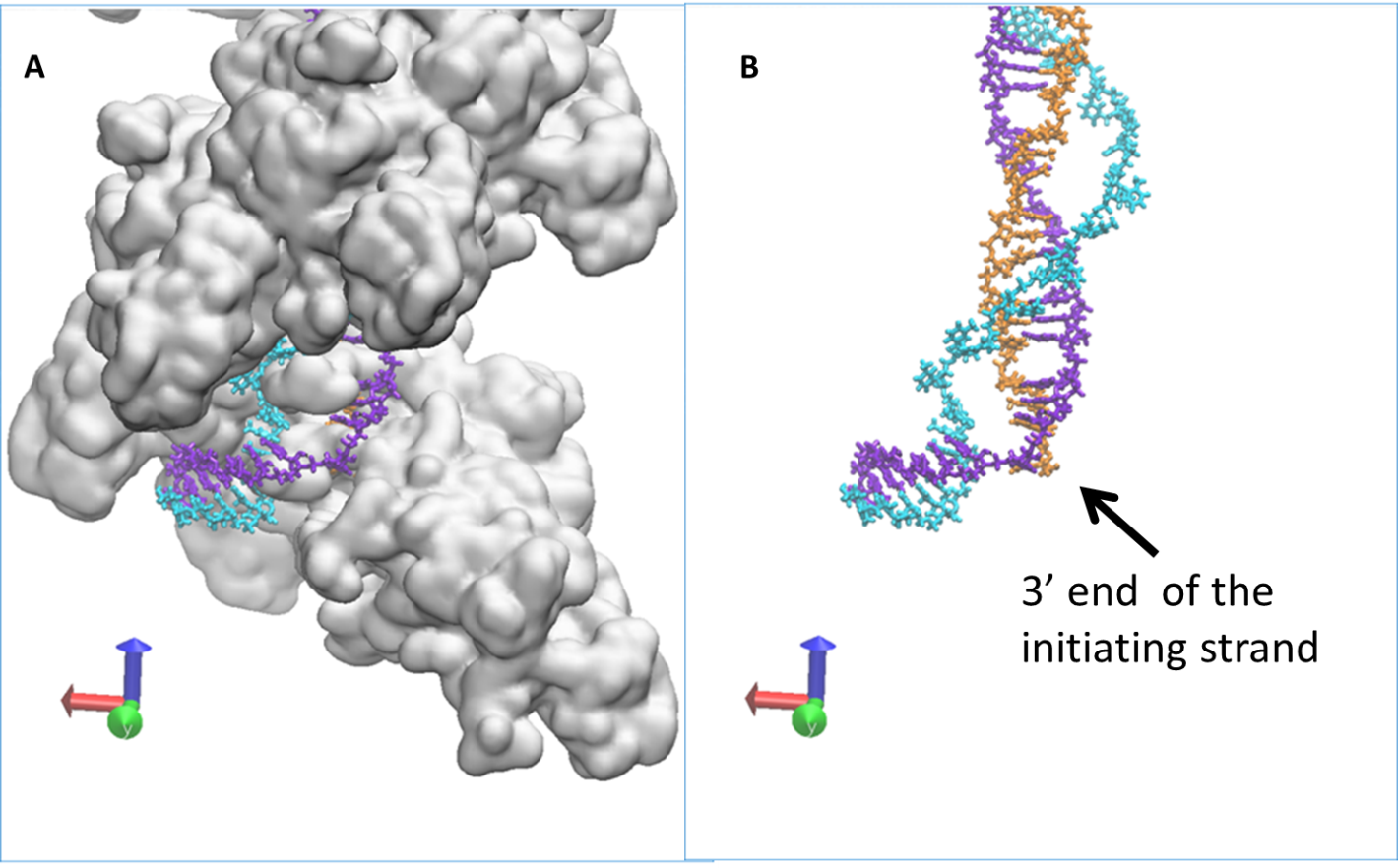


**Figure S4. Model of the RecA DNA structure in which the heteroduplex extends to the 3′ end of the initiating strand.** A, structure with the protein shown in a surface representation and the outgoing (cyan), complementary (purple), and initiating (orange) strands shown in a ball and stick representation. B, same as A, but showing only the DNA strands. The arrow points to the 3′ end of the initiating strand, highlighting the interface between the pairing of the complementary strand to the initiating strand and the pairing of the complementary strand to the outgoing strand.


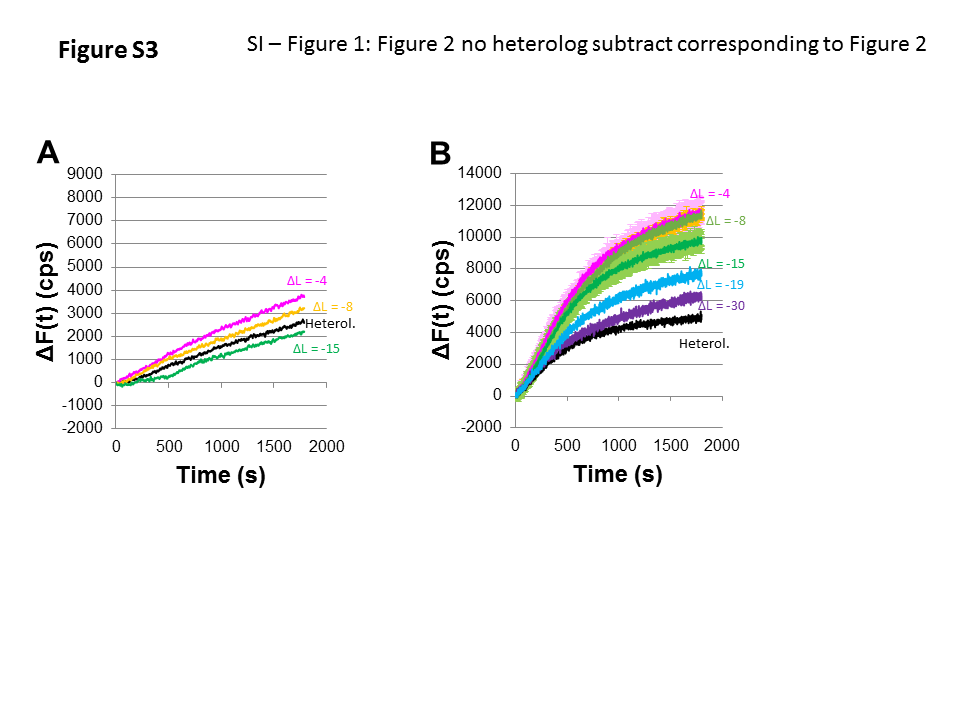


**Figure S5. Measurements of the extension of the invading strand in D-loops formed by RecA-mediated strand exchange in long dsDNA.** Schematic of the experimental design is the same as the schematic shown in Fig. 3A. A, ΔF(t) vs. time curves of strand exchange in the presence of DNA Pol IV for ΔL = -4 (magenta), ΔL = -8 (orange), and ΔL = -15 (green). This graph is completely analogous to Fig. 3B but without subtraction of the curve of the heterologous ssDNA-RecA filament results shown in black. B, analogous results in the presence of DNA LF-Bsu Pol corresponding to Fig. 3C but without the subtraction of the heterologous ssDNA results shown in black; ΔL = -4 (magenta), ΔL = -8 (orange), and ΔL = -15 (green); ΔL = -19 (blue), and ΔL = -30 (purple). Thus, the results shown in Fig. 2 may be different for the two polymerases because in the 180 bp dsDNA, DNA LF-Bsu extends the invading strand much more than DNA Pol IV.

**
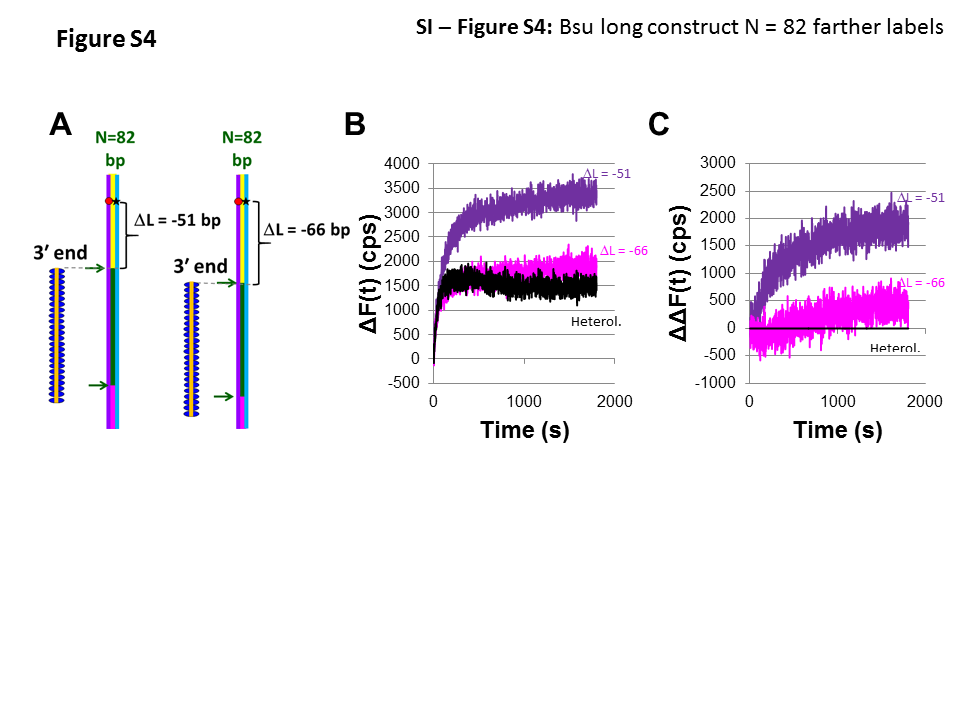
**

**Figure S6. Measurements of the extension of the invading strand in D-loops formed by RecA-mediated strand exchange in long dsDNA.** A, schematic of the experimental design using the same symbols as Fig. 2A. The dsDNA is 180 bp long, and the fluorophores are positioned at 11 bp (rhodamine) and 9 bp (fluorescein) beyond the end of the dsDNA. The 3′ ends of the N = 82 initiating strands are 62 bp and 77 bp from the end of the dsDNA, yielding ΔL = -51 and ΔL = -66. B, ΔF(t) vs. time curves of strand exchange in the presence of DNA LF-Bsu Pol for ΔL = -51 (purple) and ΔL = -66 (magenta) with the black curve representing results for the heterologous ssDNA-RecA filament. Results shown are for a single data set. C, ΔΔF(t) vs. time curves of strand exchange in the presence of DNA LF-Bsu Pol for ΔL = -51 (purple) and ΔL = -66 (magenta). The second delta indicates that the ΔF(t) curve for the heterologous ssDNA-RecA filament (i.e. N = 0) was subtracted from the ΔF(t) curves for N = 82 shown in purple and magenta in B. Results shown are for a single data set.


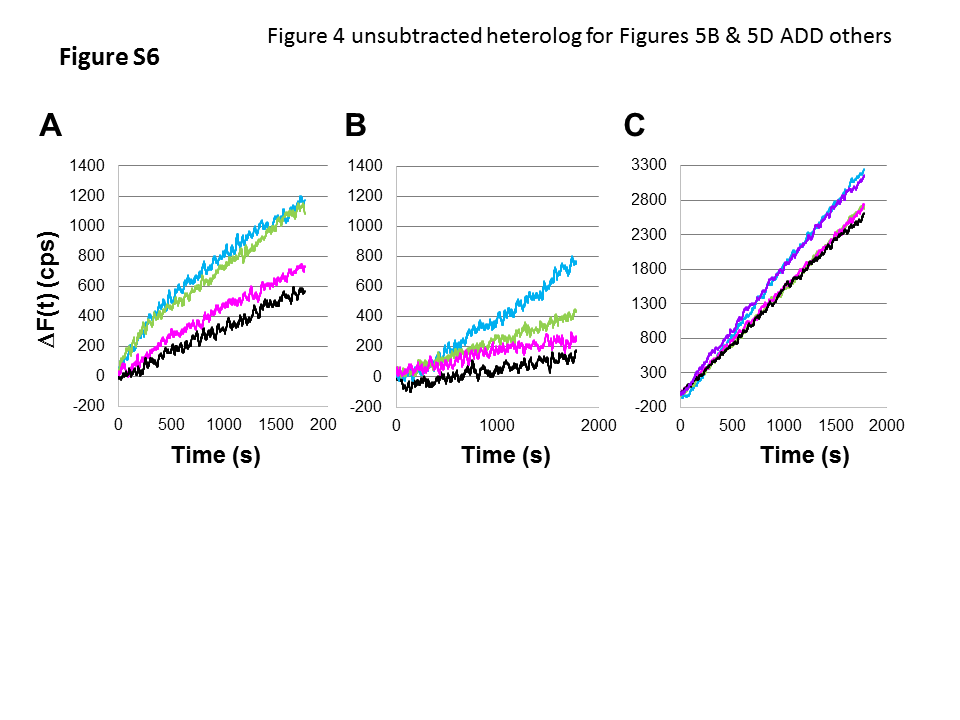


**Figure S7. Strand-exchange mediated synthesis in the presence of DNA Pol IV.** A, ΔF(t) vs. time curves for N = 82 (blue), N = 50 (green), N = 20 (magenta), and N = 0 (black) for the experimental design shown in Fig. 4A. This plot is analogous to Fig. 4D but without subtraction of the heterologous filament results. The results are for a single data set. B, ΔF(t) vs. time curves for the experimental design shown schematically in Fig. 4B for N = 82 (blue), N = 50 (green), N = 20 (magenta) and heterologous filament N = 0 (black). This is analogous to Fig. 4E but without the subtraction of any results. C, ΔF(t) vs. time curves for the experimental design shown schematically in Fig. 4C for N = 98 (purple), N = 82 (blue), N = 50 (green), N = 20 (magenta), and heterologous filament N = 0 (black). This is analogous to Fig. 4F but without the subtraction of any results. Results are for a single data set.


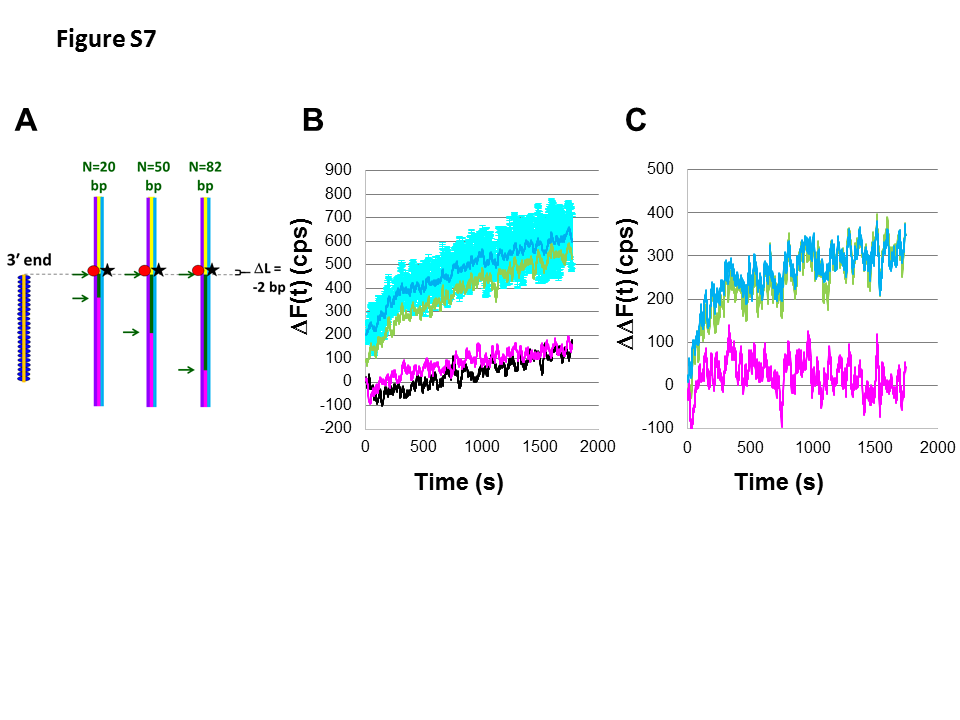


**Figure S8. Strand-exchange mediated synthesis in the presence of DNA Pol IV.** A, schematic of the experimental design using the same symbols as in Fig. 4A. The dsDNA is 180 bp long, and the fluorophores are 58 (rhodamine) and 57 bp (fluorescein) from the end of the dsDNA. The 3′ end of the ssDNA-RecA filaments is 60 bp from the end of the dsDNA, yielding ΔL = -2. B, ΔF(t) vs. time curves for N = 82 (blue), N = 50 (green), N = 20 (magenta), and N = 0 (black) for the experimental design shown in A. The results are for a single data set. C, ΔΔF(t) vs. time curves for the experimental design shown in A for N = 82 (blue), N = 50 (green), and N = 20 (magenta). The second delta indicates that the fluorescent signal of the heterologous ssDNA-RecA filament (N = 0) has been subtracted from each curve. The results are for a single data set.


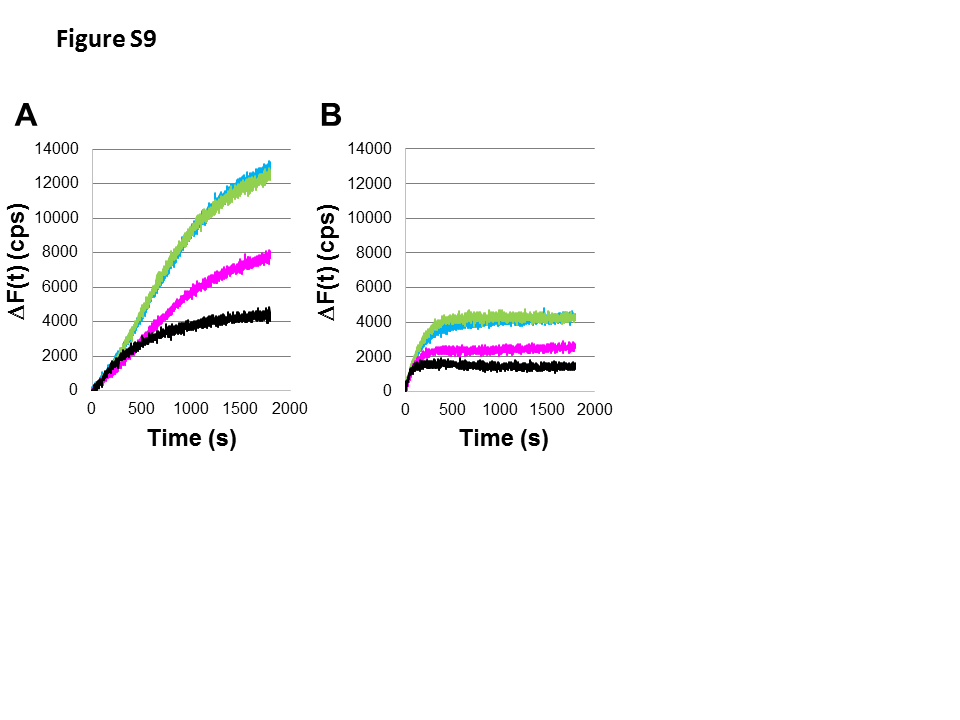


**Figure S9. Strand-exchange mediated synthesis in the presence of DNA LF-Bsu Pol.** A, ΔF(t) vs. time curves for ΔL = -1. The experimental schematic is shown in Fig. 4A. This plot is analogous to Fig. 5A but without subtraction of the heterologous filament curve. B, ΔF(t) vs. time curves for ΔL = -48. This plot is analogous to Fig. 5C but without subtraction of the heterologous curve. Schematic for this experiment is shown in Fig. 5B.
